# Efficacy of three-dimensional roadmapping by fusion of computed tomography angiography with volumetric data from an angiography machine in endovascular therapy for iliac chronic total occlusion: a case report

**DOI:** 10.1186/s42155-019-0076-y

**Published:** 2019-10-21

**Authors:** Naoki Hayakawa, Satoshi Kodera, Noriyoshi Ohki, Junji Kanda

**Affiliations:** 1 Department of Cardiovascular Medicine, Asahi General Hospital, I-1326 Asahi, Chiba, 289-2511 Japan; 2 Department of Cardiovascular Medicine, University of Tokyo Hospital, Tokyo, Japan; 3 Department of Radiology, Asahi General Hospital, Chiba, Japan

**Keywords:** Endovascular therapy, Three-dimensional roadmap, Fusion imaging, Chronic total occlusion

## Abstract

**Background:**

The usefulness of endovascular therapy (EVT) for the iliac artery has been established. However, difficult cases such as a long total occlusion and tortuous vessels are sometimes encountered. We recently performed rotational angiography with an angiography machine immediately before EVT and fused three-dimensional (3D) anatomical information obtained from preoperative enhanced computed tomography (CT) that had been performed in advance to create a 3D roadmap. We termed this method the CT fusion 3D roadmap (CTf3D-RM) technique and used it for treatment of iliac occlusive disease.

**Case presentation:**

A 73-year-old man presented with pain in his left leg while resting. CT showed total occlusion from the ostium of the common iliac artery (CIA) to the distal part of the external iliac artery (EIA). A guiding sheath was inserted from the left common femoral artery using the CTf3D-RM technique, and the occlusive vessel was clearly observed. The guidewire could be passed retrogradely without bidirectional wiring. The time taken to pass the guidewire was only about 9 min despite the long and hard chronic total occlusion (CTO). Intravascular ultrasound showed that all of the guidewire followed the intraplaque route. After ballooning the entire lesion, we deployed two stent grafts and three bare nitinol stents from the left CIA ostium to the distal EIA. Final angiography showed good expansion and sufficient flow to the left leg.

**Conclusions:**

The use of a 3D roadmap by fusion of CT angiography with volumetric data from an angiography machine in EVT for iliac CTO was shown to be effective.

## Background

The usefulness of endovascular therapy (EVT) for the iliac artery has been established, and high treatment success rates and low complication rates have been achieved (Yamauchi et al. 2019). However, difficult cases such as a long total occlusion and tortuous vessels are sometimes encountered. Treatment of chronic total occlusion (CTO) is difficult because the occlusive vessel cannot be seen by angiography. Several imaging-guided wiring techniques such as intravascular ultrasound (IVUS) have been reported; however, each method has weaknesses associated with technical difficulty and the inherent limitations of the devices (Kawasaki et al. 2008). We recently performed rotational angiography with an angiography machine immediately before EVT and fused three-dimensional (3D) anatomical information obtained from preprocedural enhanced computed tomography (CT) that had been performed in advance to create a 3D roadmap including virtual occlusive vessels. We named this method the CT fusion 3D roadmap (CTf3D-RM) technique. It is possible to understand the correct position of the guide wire in patients with CTO in real time using a 3D roadmap. We herein report a case of successful treatment of iliac CTO using the CTf3D-RM technique.

## Case report

A 73-year-old man with progressive pain during rest and cyanosis of his left lower limb was referred to our institution for revascularization. At presentation, the patient had hypertension, atrial fibrillation, chronic heart failure, and old cerebral infarction. The patient’s ankle–brachial index was 0.39 on the left side and 1.01 on the right. Enhanced CT revealed total occlusion from the ostium of the left common iliac artery (CIA) to the distal end of the external iliac artery (EIA) and total occlusion of the left superficial femoral artery.

**Fig. 1:**
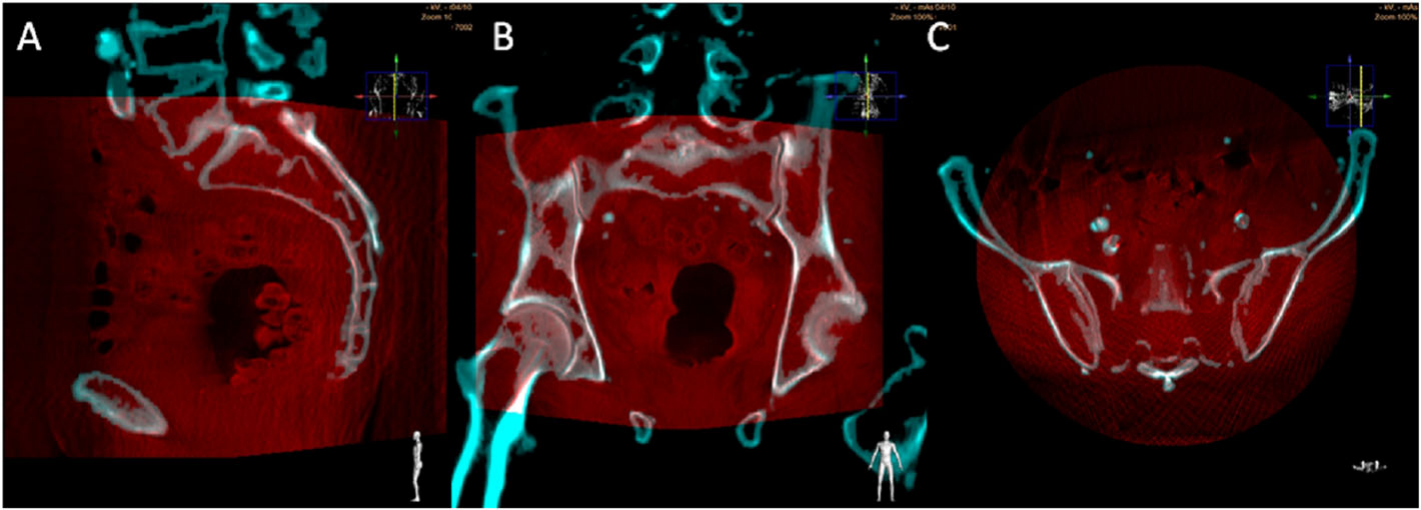
3D roadmap was created by fusing CT data with virtual vessels and the actual angiography image using the positions of the bones (**a**: sagittal planes, **b**: coronal planes, **c**: axial planes). Blue zone was from pre procedural CT data. And red zone was the pelvic region with 3D rotational angiography using an angiography machine and reconstructed the acquired volume data

**Fig. 2:**
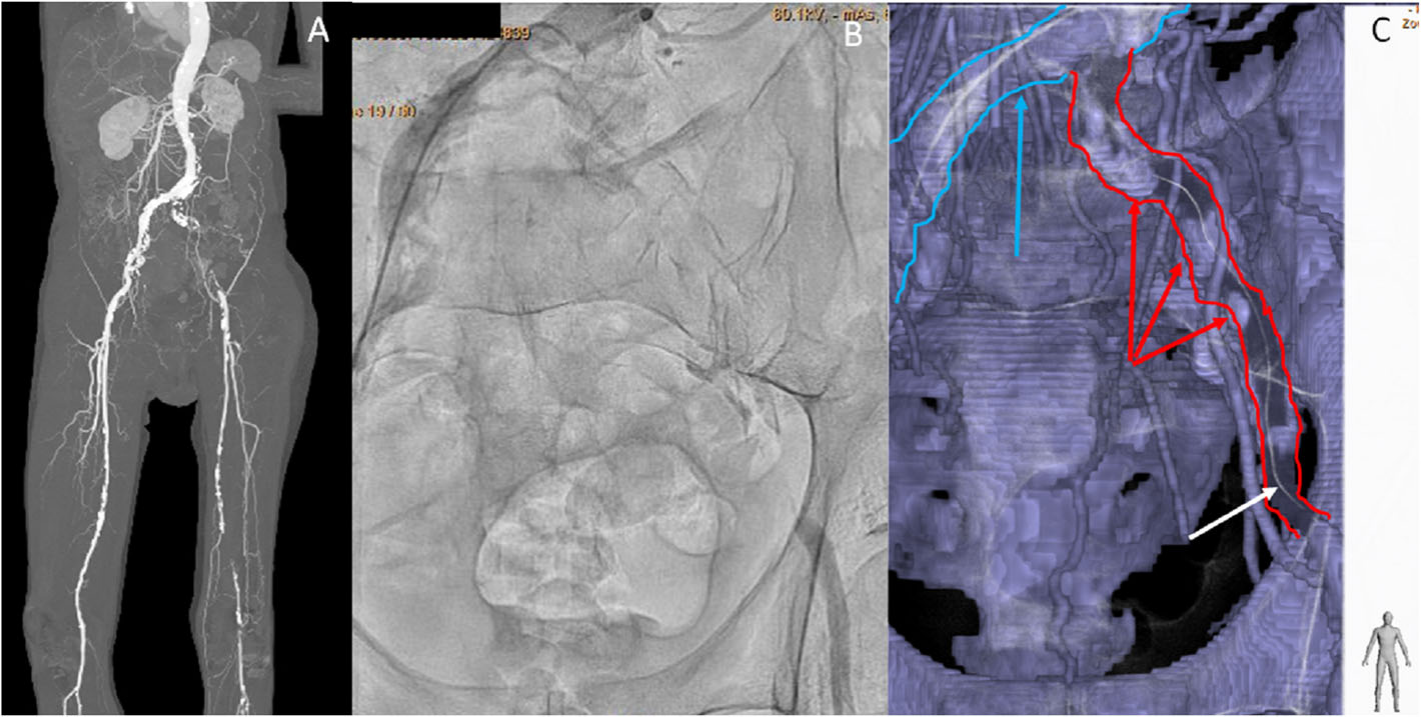
**a**: Pre procedural enhanced CT revealed total occlusion from the ostium of the left common iliac artery (CIA) to the distal end of the external iliac artery (EIA) and total occlusion of the left superficial femoral artery. **b**: Bidirectional control angiography revealed total occlusion from the ostium of the left CIA to the distal aspect of the left EIA. **c**: This is CTf3D-RM (blue arrow showed opened aorta and right CIA, red arrow showed virtual occluded vessels, white arrow showed guidewire). The control angiography and CTf3D-RM images matched well

**Fig. 3:**
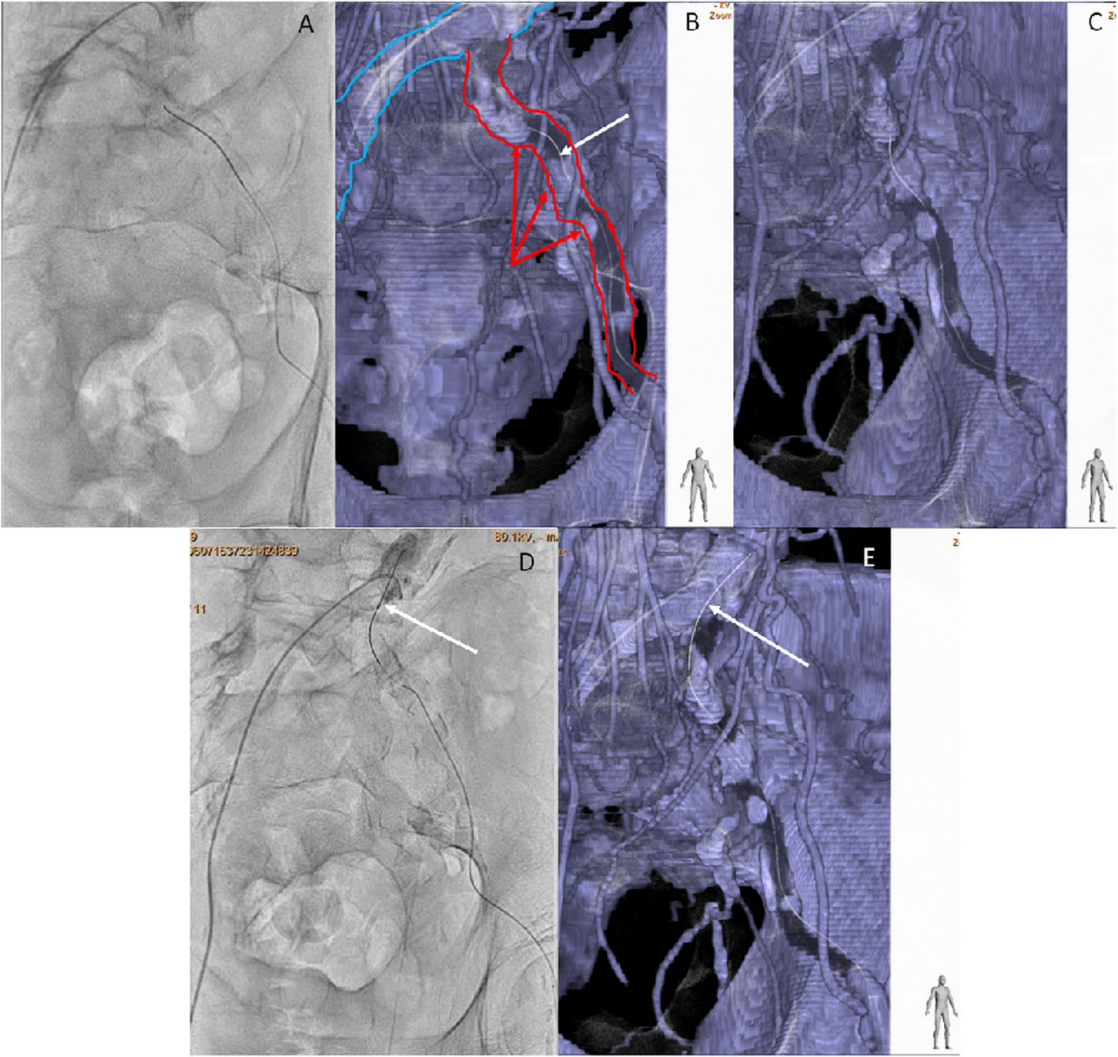
**a** and **b**: We advanced the guidewire while aiming at the center of the virtually occluded iliac artery on the CTf3D-RM image (blue arrow showed opened aorta and right CIA, red arrow showed virtual occluded vessels, white arrow showed guidewire). **c**: We checked the wire position in RAO view. The guide wire was almost centor in the virtual occluded iliac artery. **d** and **e**: We could pass the guidewire retrogradely into the aorta

**Fig. 4:**
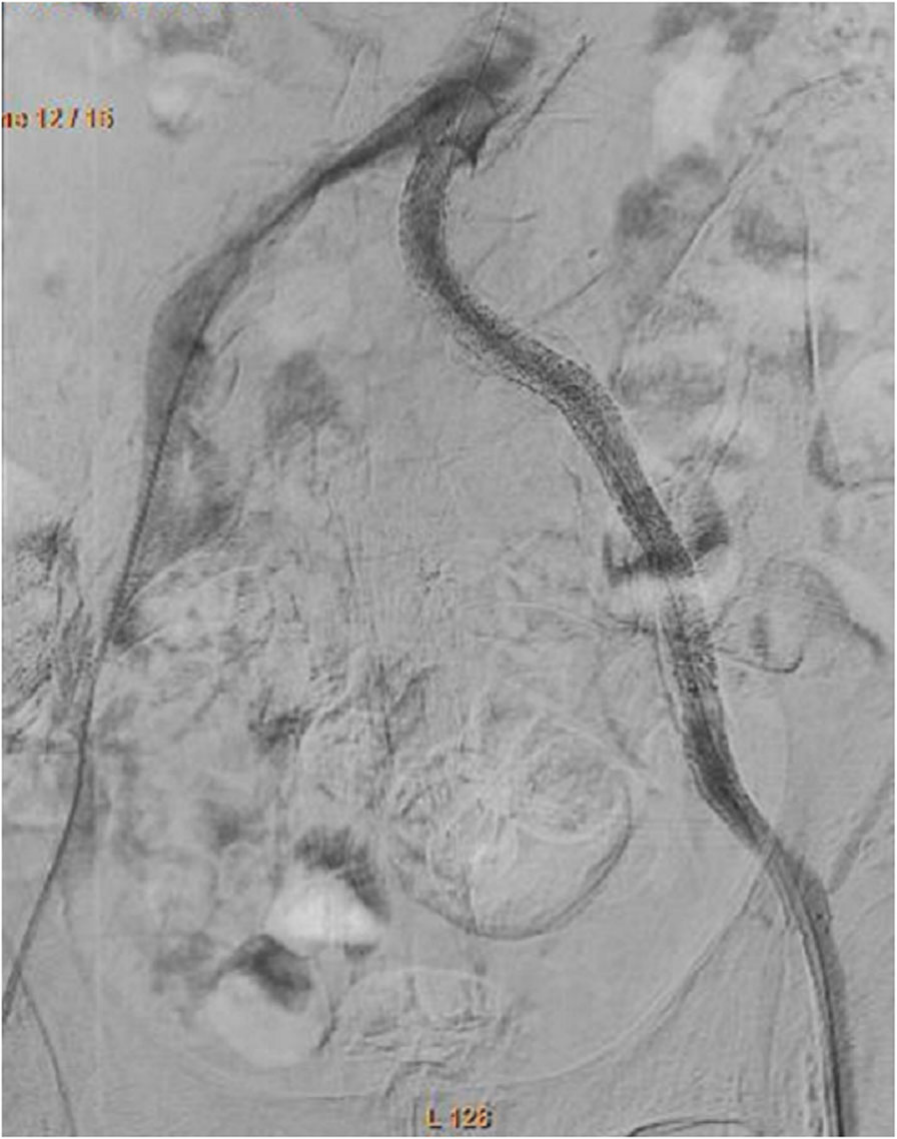
Final angiography showed a good result

We attempted to treat the CTO of the left iliac artery. We decided to perform the CTf3D-RM technique. Angiography was performed with an AZURION 7M20C machine (Philips, Amsterdam, The Netherlands) and a SYNAPSE VINCENT computer workstation (FUJIFILM, Tokyo, Japan). The 3D roadmap was created as follows. First, our radiation technical physician constructed a virtual occlusive vessel by analyzing the central line of the occluded vessel to be treated with curved multiplanar reconstruction images obtained from thin-slice data of the arterial phase. Next, we examined the pelvic region with 3D rotational angiography using an angiography machine and reconstructed the acquired volume data. Finally, we fused the CT data with the virtual vessels and the actual angiography image using the positions of the bones (Fig. 1). The patient’s body was fixed using a VacLoc® device (Toyo Medic Co., Tokyo, Japan), which can fix the patient’s position using negative-pressure aspiration. The CTf3D-RM can follow even changing the flat panel inch size, panning, angle, and magnification. After creating the 3D roadmap, we inserted a Parent Plus 60® guiding sheath (Medikit Co., Tokyo, Japan) by aiming it at the distal part of the left common femoral artery as a guide. We then inserted a 5-Fr short sheath in the right common femoral artery. Bidirectional control angiography revealed total occlusion from the ostium of the left CIA to the distal aspect of the left EIA (Fig. 2b). The control angiography and CTf3D-RM images were matched as much as possible (Fig. 2a-c). We used a Halberd® guidewire (Asahi Intecc Co., Aichi, Japan) and a Corsair Armet® microcatheter (Asahi Intecc Co.) in a retrograde manner. We advanced the guidewire while aiming at the center of the virtually occluded iliac artery on the CTf3D-RM image (Fig. 3a-e). The lesion was very hard, but we successfully passed the guidewire retrogradely into the aorta. Although the lesion was Trans-Atlantic Inter-Society Consensus (TASC) type D, it took only about 9 min to pass the guidewire. Furthermore, IVUS showed that all of the guidewire followed the intraplaque route. After ballooning, we deployed a Viabahn VBX 8.0- × 59-mm® stent graft (GORE, Tokyo, Japan), a Viabahn VBX 8.0- × 29-mm® stent graft (GORE), a SMART 9.0- × 60-mm® stent (Cardinal Health Japan, Tokyo, Japan), and a SMART 9.0- × 60-mm® stent (Cardinal Health Japan) from the CIA ostium to the distal EIA. Because a thrombus was found at the CIA ostium due to plaque shift, a SMART 10.0- × 40-mm® stent (Cardinal Health Japan) was added to the immediately proximal part of the CIA to seal the protruded thrombus. After ballooning the entire lesion, final angiography showed good expansion and sufficient flow to the left leg (Fig. 4). The dose–area product was 17.0 Gy·cm^2^. The total fluoroscopy time was 47.5 min. The patient’s ankle–brachial index was still 0.59 because residual lesions were present in his left superficial femoral artery and below the knee arteries. However, his pain during rest and cyanosis were dramatically improved after the procedure.

## Discussion

Even with a TASC type C/D lesion, acceptable results of EVT have been reported in patients with aortoiliac disease (Yamauchi et al. 2019; Leville et al. 2006). Even for a very long total occlusion, once the guidewire is passed, a good result can be obtained by stent or stent graft implantation; the passage of the guidewire is very important. Additionally, because a fatal outcome due to vascular perforation or injury is possible in patients with aortoiliac lesions, the guidewire should be passed through the most optimal route possible. Various methods for passing the guidewire to a CTO lesion have been reported, including IVUS guidance, echo-Doppler (duplex) guidance, intravenous ultrasound guidance, bidirectional wiring, and subintimal angioplasty (Kawasaki et al. 2008; Schmidt et al. 2012; Kawarada et al. 2010; Hishikari et al. 2015). However, no gold standard method has been established because each method has its weaknesses.

The CTf3D-RM method does not require a special technique or device to perform the CTO intervention. This method involves highly objective guidewire manipulation that is secured by advancing the guidewire according to the virtual occluded vessel displayed on the angiographic image. The CTf3D-RM can follow changes in the flat panel inch size, panning, tube angle, and magnification. Thus, there is no need to create a new roadmap each time, and the procedure can be continued using the previously created roadmap. This method may contribute to shortening the procedure time, decreasing the number of guidewires needed, and reducing the amount of contrast medium used. Although the lesion in the present case was very complex, the time required to pass the guidewire was as short as 9 min, and the procedure could be completed without using an extra guidewire. In addition, the whole image of the vessel can be obtained, which is very helpful when choosing the puncture site of the femoral artery or when placing a stent.

Several reports have described the feasibility of image fusion of preoperative multidetector CT, cone-beam CT, or magnetic resonance angiography with intraprocedural fluoroscopy for creation of a roadmap during EVT and neurointervention (Jones et al. 2018; Sailer et al. 2015; Ierardi et al. 2016; Schwein et al. 2017). These reports suggested that it is easy to obtain the whole image of the vessel of interest, which can contribute to safety by reducing contrast agent use and radiation exposure (Schwein et al. 2017). Certainly, contrast-induced nephropathy is problematic for patients with chronic kidney disease, and although several effective methods have been reported, it seems that the CTf3D-RM technique can serve as another such method (Fujihara et al. 2015; Hayakawa et al. 2019; Mariani Jr et al. 2014). A major limitation of this fusion roadmap technique seems to be patient movement and image artifact. Other problems include different patient positions during preoperative CT or magnetic resonance angiography and interventional procedures. According to one study, a mismatch of several millimeters may not be clinically significant in aortoiliac lesions (Ierardi et al. 2016). Additionally, in previous reports, roadmaps were characterized by drawing opened vessels. These reports were mainly intended to create roadmaps for opened vessels to place wires and devices without using contrast agents. However, the most important point of our method is to visualize the details of the occluded vessel, mainly using the creation of a 3D roadmap as a support tool in precise wiring for the CTO lesion. Therefore, our method emphasizes the construction of a virtual vessel based on an accurate tracing of the central line of the occluded vessel and accurate reconstruction of the posture during the preoperative CT imaging. The difference between our method and those described in previous reports is that our method is not mainly intended to treat opened vessels, but rather to assist the passage of guidewires within the CTO by accurately depicting the occluded vessel and creating virtual occluded vessel. In the present case, as confirmed by IVUS after crossing through the guidewire, all of the guidewire passed through the intraplaque route. Although the posture and vessel course may change and the roadmap may shift because of the prolonged procedure time, it is relatively easy to readjust the roadmap using bone and artery calcification. The iliac artery is located within the pelvis and is relatively unaffected by changes in position; however, it is convenient to use a fixation device for long-term position fixation. The procedure can be performed without using the fixation device; in such cases, however, it may be necessary to correct the deviation between the fusion image and the angiography image more frequently. We believe that accuracy of the CTf3D-RM technique depends on postural fixation of the patient during the procedure and constant correction of image shifts using the location of vessel calcifications and bones. Even if this method is used, the image may shift, in which case it is desirable to refer to another modality such as IVUS.

## Conclusions

We have herein reported a case of CTO of the iliac artery for which the CTf3D-RM technique was successfully used. The creation of an accurate 3D roadmap allowed for accurate and rapid passage of the guidewire, even in this case of a long total occlusion.

## Data Availability

Not applicable.

## Abbreviations

3D: three-dimensional
CIA: common iliac artery
CT: computed tomography
CTf3D-RM: computed tomography fusion three-dimensional roadmap
CTO: chronic total occlusion
EIA: external iliac artery
EVT: endovascular therapy
IVUS: intravascular ultrasound
TASC: Trans-Atlantic Inter-Society Consensus
